# IgA nephropathy with minimal change disease associated with primary Sjögren’s syndrome: A case report

**DOI:** 10.1097/MD.0000000000033892

**Published:** 2023-06-02

**Authors:** Jungyoon Choi, Tae Won Lee, Eunjin Bae, Ha Nee Jang, Hyo Jung An, Se-Ho Chang, Dong Jun Park

**Affiliations:** a Department of Internal Medicine, Gyeongsang National University Changwon Hospital, Changwon, South Korea; b Department of Internal Medicine, Gyeongsang National University College of Medicine, Jinju, South Korea; c Institute of Health Science, Gyeongsang National University, Jinju, South Korea; d Department of Internal Medicine, Gyeongsang National University Hospital, Jinju, South Korea; e Department of Pathology, Gyeongsang National University Changwon Hospital, Changwon, South Korea; f Department of Pathology, Gyeongsang National University College of Medicine, Jinju, South Korea.

**Keywords:** IgA nephropathy, minimal change disease, proteinuria, Sjögren’s syndrome

## Abstract

Only 1 case of IgA nephropathy (IgAN) with minimal change disease (MCD) associated with primary Sjögren’s syndrome (SS) has been reported. We additionally describe IgAN with MCD associated with primary SS.

**Patient concerns:**

A 80-year-old woman visited our hospital complaining of generalized edema that had started 4 weeks prior. She reported a sense of thirst and dry eye for the last 5 years.

**Diagnoses:**

Her initial laboratory findings were compatible with nephrotic syndrome; both the antinuclear antibody (1:80) and anti-SS-A (Ro) antibody (200 U/mL) tests were positive. A salivary gland scan revealed markedly decreased uptake for both the parotid and submandibular glands. The Schirmer test was positive. The random urine protein/creatinine ratio was 10 mg/mg. Renal biopsy was compatible with IgAN with superimposed MCD.

**Interventions:**

Furosemide was intravenously administered with intermittent albumin infusion for her edema control. She was started on prednisone 40mg daily for 6 weeks, which was tapered to 5 mg for another 6 months after starting prednisolone.

**Outcomes:**

Over the next 6 months, her edema improved and the proteinuria decreased significantly.

**Lessons:**

Physician should suspect IgA with MCD when patient with SS clinically showed nephrotic syndrome, and perform renal biopsy for pathologically diagnosis and appropriate treatment.

## 1. Introduction

Sjögren’s syndrome (SS) is a chronic autoimmune inflammatory disorder characterized by B- and T-cell responses to autoantigens that causes lymphocytic infiltration of the epithelial tissues of exocrine glands (mainly the lacrimal and salivary glands).^[1]^ However, extraglandular manifestations of SS develop in up to 25% of patients and can compromise organ function and even cause death.^[2]^ Patients may experience severe interstitial lung disease, cutaneous vasculitis, peripheral neuropathy, and/or hematological complications such as lymphoma.^[3]^ Renal involvement is a life-threatening extraglandular manifestation and presents in various forms, including tubulointerstitial nephritis (TIN), membranoproliferative glomerulonephritis (GN), mesangial proliferative GN, membranous nephropathy, and occasionally IgA nephropathy (IgAN).^[4]^ TIN remains the most common presentation^[4]^ and is often characterized by distal (type I) renal tubular acidosis ^[5]^; the acidosis is less commonly proximal (type II).^[6]^ Although a few reports on the co-existence of IgAN and SS^[7–9]^ have appeared, only 1 case of IgAN with minimal change disease (MCD) (an unusual form of IgAN) associated with SS has been described.^[10]^ We report the second SS patient with IgAN and MCD.

## 2. Ethical statement and consent

Written informed consent was obtained from the patient and her son for publication of their case report and any accompanying images. The study protocol was approved by the Institutional Review Board of Gyeongsang National University Changwon Hospital (IRB no. 2022-11-022).

## 3. Case presentation

An 80-year-old Korean female visited our hospital complaining of generalized edema that had started 4 weeks prior. Her medical history included anemia, hypertension, and hypothyroidism, and her medications included ferrous sulfate 512 mg, losartan 100 mg, and levothyroxine 0.05 mg daily. She stated that she had suffered from a severe dry mouth and dry for the last 5 years. However, she had not reported these symptoms to medical staff. She strongly denied the use of toxins, drugs, and dietary supplements, and stated that she took only the medicines described. She denied smoking and alcohol consumption. She lacked any upper respiratory tract symptom or sign; there was no cough, sore throat, fever, or rhinorrhea. She claimed that she had gained 4 kg over the past 3 months. She asserted she had never heard of urine abnormalities.

**Figure 1.**
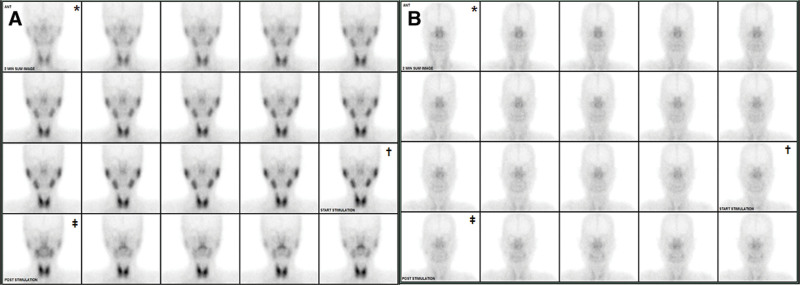
(A) Salivary scan (Tc-99mO4-) showing normal uptake of both parotid and submandibular glands in a healthy 40-year-old woman. (B) Salivary scan showing severely decreased uptake of both parotid and submandibular glands in our patient. *Two minutes after intravenous tracer liquid infusion. **†**Half-hour after intravenous tracer liquid infusion. Three minutes after sialagogue intake.

Her initial vital signs were as follows: blood pressure, 133/73 mm Hg, heart rate, 86 beats/minutes. respiratory rate, 20 breaths/minutes. and body temperature, 36.7℃. She had a puffy face and peri-orbital edema. The conjunctiva was anemic but the sclera was not icteric. Neck examination did not reveal an enlarged thyroid or palpable lymphadenopathy. No skin lesion was found anywhere on the body. Three regions of pretibial pitting edema were found on both legs. Her initial complete blood count, biochemical findings, urinalysis with microscopic findings are shown in Table 1. Her anemia was presumed to be caused by her chronic disease (Table 1). The random urine protein/creatinine ratio (PCR) was 10 mg/mg (range: 0–0.2 mg/mg). Further workup including thyroid function, hepatitis B-and C serology as well as immunoglobulins were normal. The serum C3 and C4 levels were 134.3 mg/dL (range: 90–180 mg/dL) and 44.9 mg/dL (range: 10–40 mg/dL), respectively. The antinuclear antibody was positive (homogeneous pattern; 1:80) but the anti-ds DNA antibody immunoglobulin G (IgG) and immunoglobulin M (IgM) tests were negative. The anti-Ro antibody test was positive (200 IU/mL; range: 15–25 IU/mL) but the anti-La antibody test was negative (3.8 IU/mL; range: 15–25 IU/mL). anticardiolipin IgG and IgM antibody tests were negative, as was the anti-Beta 2 glycoprotein I antibody test. The Schirmer test was strongly positive. A salivary scan using Tx-99m pertechnetate (10 mCi) revealed severely decreased uptake by both the parotid and submandibular glands (Fig. 1).

**Table 1:** Serum and urine laboratory findings.

Complete blood count (CBC)	
WBC (4.0–10.0 × 10^9^/L)	7.1 × 10^9^/L
Neutrophil (50–75%)	86.8%
Lymphocyte (20–44%)	9.2%
Monocyte (2–9%)	3.7%
Eosinophil (1–5%)	0.3%
Hemoglobin (12–16 g/dL)	8.1 g/dL
Hematocrit (36–48%)	25 %
MCV (79–95 fL)	97 fL
Platelet (130–400 × 10^9^/L)	361 × 10^9^/L
Reticulocyte count (0.5–2.0 %)	2.1%
Biochemical findings	
ALP (30–120 IU/L)	133 IU/L
AST (1–37 IU/L)	34 IU/L
ALT (0–41 IU/L)	42 IU/L
Glucose (70–110 mg/dL)	123 mg/dL
Protein (6.6–8.7 g/dL)	5.2 mg/dL
Albumin (3.5–5.2 g/dL)	2.4 mg/dL
Cholesterol (120–200 mg/dL)	306 mg/dL
BUN (8.0–20.0 mg/dL)	22.7 mg/dL
Creatinine (0.51–0.95 mg/dL)	0.59 mg/dL
Sodium (135–145 mmol/L)	138 mmol/L
Potassium (3.3–5.1 mmol/L)	4.6 mmol/L
Chloride (98–110 mmol/L)	105 mmol/L
Iron profile workup	
Iron (60–180 *u*g/dL)	63 *u*g/dL
TIBC (230–430 *u*g/dL)	176 *u*g/dL
TSAT (20–55 *u*g/dL)	35.8%
Ferritin (10–291 ng/mL)	515.9 ng/mL
Urinalysis with micro	
SG (1.005–1.030)	1.011
Protein (−)	(+++)
Blood (−)	(+)
RBC count (0–4/high-power field)	10–19/HPF

ALP = alkaline phosphatase, ALT = alanine aminotransferase, AST = aspartate aminotransferase, BUN = blood urea nitrogen, HPF = high-power field, MCV = mean cell volume, RBC = red blood cell, SG = specific gravity, TIBC = total iron binding capacity, TSAT = threshold transferrin saturation, WBC = white blood cell.

**Figure 2.**
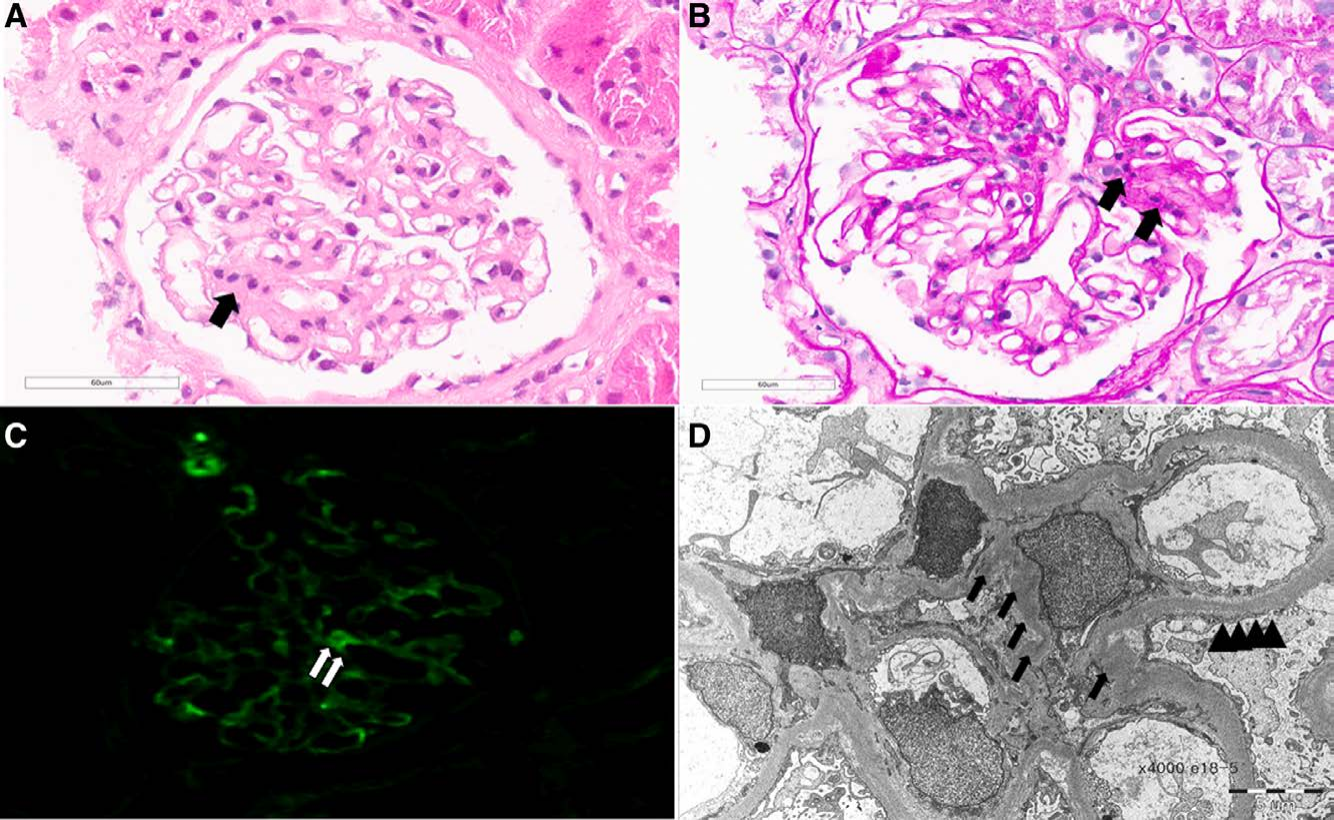
(A) Hematoxylin and Eosin (HE). (B) Periodic acid-Schiff (PAS) staining showing increased mesangial cellularity and expansion (x400). (C) Immunofluorescence staining showing mesangial deposits of IgA (+) (x400). (D) Electron microscopy showing electron-dense deposit on the mesangium (arrow) and diffuse foot process effacement (arrow head) (x4000).

**Figure 3.**
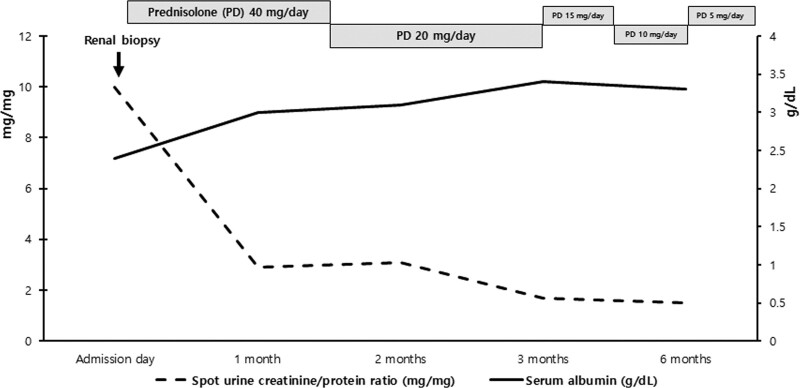
The change of serum albumin level and random urine protein/creatinine ratio.

Furosemide was intravenously administered with intermittent albumin infusion for her systemic edema control. Artificial tears were applied every few hours for her dry eyes. Five milligrams of pilocarpine were initially prescribed and the dose was increased to 10 mg per day for her dry mouth. On the second day of admission, renal biopsy was performed to evaluate renal pathology. Of 16 glomeruli seen under a light microscope, one was globally sclerotic. Eleven of fifteen glomeruli exhibited increased cellularity and mesangial lesions. The biopsy also revealed mild focal tubular atrophy and lymphocytic infiltration. Immunofluorescence studies revealed the mesangial deposit status: IgA (+), IgG (−), IgM (+), C3 (+), C4 (−), and C1q (−). Immune-complex electron-dense deposits were noted in the glomerular mesangium, but not in the subendothelial area; electron microscopy (EM) revealed diffuse podocyte foot effacement (Fig. 2). The findings were consistent with a diagnosis of IgA nephropathy (Oxford classification; M1, E0, S0, T1, and C0) with MCD. Her dry eye and mouth have been improved a little. On the 5th day of admission, she was started on prednisone 40 mg daily for 6 weeks, which was tapered to 20 mg for another 12 weeks. In the 4 weeks after steroid and diuretic therapy, the random urine PCR decreased to 2.6 mg/mg, the serum albumin level increased to 3 g/dL. Urine PCR further decreased to 1.7 mg/mg at 12 weeks and remained unchanged thereafter (Fig. 3). Prednisolone dose has been reduced to 5 mg 6 months after starting prednisolone. The patient has been followed for 1 year on 5 mg maintenance of prednisolone without significant side effects. Her edema has been disappeared and sicca symptoms have been improved, but persisted

## 4. Discussion

We described a case of IgAN with MCD associated with SS. The proteinuria recovered to nearly normal levels after 3 months of steroid therapy. Primary SS accompanied by IgAN with MCD should be viewed as another form of renal involvement associated with primary SS.

The prevalence of renal involvement in SS patients varies. One cross-sectional study found that 27% of SS patients exhibited frank abnormalities suggesting a tubular or glomerular pathology^[11]^; renal biopsies revealed tubulointerstitial nephritis in 6 patients and glomerular disease in 3 others. Another retrospective study assessed 7276 SS patients over a 40-year period and analyzed 24 who underwent renal biopsy.^[3]^ Seventeen patients had acute or chronic TIN, 2 had cryoglobulinemic GN, and 2 others had focal segmental glomerulosclerosis. A third study included 471 patients followed-up for 10 years; 18 underwent kidney biopsies.^[12]^ Glomerular histological examination revealed changes compatible with membranoproliferative glomerulonephritis in 5 patients and proliferative mesangial GN in 4 others. However, renal biopsy in the studies above did not identify IgAN.

A few cases of IgAN associated with SS have been reported. However, their renal biopsy would not reveal foot process effacement in EM whereas mesangial IgA deposits existed in all cases.^[7–9]^ Only 1 case of IgAN with MCD associated with SS has been described in the literature. Mon et al^[10]^ reported a case of patient showing nephrotic syndrome, elevated serum CA19-9, and mesangial IgA deposits in renal biopsy. However, their case report lacks EM data and therefore it is unsure whether this patient really had foot process effacement. Therefore, our case is unique in that it is the first to prove foot process effacement in EM and mesangial IgA deposits restricted to mesangium in patient with SS. Other differences from Mon et al^[10]^ are the response to steroid treatment. Their patient had complete remission of proteinuria to steroid treatment, whereas our patient showed a significant reduction but still has some residual proteinuria. We cannot accurately explain the difference in the treatment response.

The pathogenesis of IgAN associated with SS is poorly understood. SS may be accompanied by dysregulation of IgA production, characterized by increased serum levels of polymeric IgA or immune complexes that are deposited in the kidney. Pathogenetic factors may include the polyclonal hypergammaglobulinemia and hypocomplementemia (especially C4) associated with SS.^[8]^ Mon et al^[10]^ suggested that circulating IgA-containing immune complexes secreted by activated monoclonal B lymphocytes are deposited in the kidney. In addition to B lymphocyte hyperactivity, dysregulation of polymeric IgA production may be a key pathogenetic factor when SS and IgAN co-occur.^[8]^ Watanabe et al^[13]^ proposed that co-occurring SS and IgAN had a genetic basis. However, our patient did not have hypergammaglobulinemia, hypocomplementemia, or a relevant family history. We cannot completely exclude the coincidental development of IgAN and MCD on SS. As IgAN with MCD are uncommon itself, it has been usually presented as case reports or in small series.^[14]^ Therefore, the question occurs whether IgAN and MCD are distinctly separate entities or if there is a component of shared pathophysiology. The characteristics of our case lies in the presence of 3 pathologies in our case; IgAN, MCD, and primary SS. Because of these characteristics, it would be better to think that these 3 components were caused by the same pathological mechanism rather than coincidence.

Patients with IgAN typically present with both hematuria and non-nephrotic proteinuria. Nephrotic proteinuria is uncommon and, if present, is usually associated with severe histological features including endocapillary proliferation, segmental sclerosis, and crescent formation linked to subendothelial IgA deposits.^[14]^ One subset of patients with IgAN who present with nephrotic proteinuria show only mild mesangial proliferative IgAN on histological examination. In such patients, EM typically reveals diffuse foot effacement without immune deposits on the peripheral capillary walls, which is reminiscent of MCD.^[15–17]^ Such patients usually respond to corticosteroids. Kidney biopsy is essential to distinguish these 2 groups.^[14]^ If IgA deposition is limited to the mesangium, endocapillary proliferation, segmental sclerosis, and crescent formation are not shown, and diffuse podocyte foot effacement are apparent on EM, the data suggest IgAN with MCD, as in our case.

No systemic immunosuppressive medication has proven effective in patients with primary SS; hydroxychloroquine or methotrexate is the mainstay treatment for uncomplicated SS.^[2,3]^ However, treatment of glomerular disease in SS patients varies according to the lesional histology.^[2]^ Almost all patients with primary SS and GN enrolled in large retrospective studies received corticosteroids, and renal function and proteinuria improved.^[2–4]^ Corticosteroid is the first-line treatment for IgAN with MCD; all patients achieve stable or improved renal function during follow-up.^[14–17]^ Our patient responded well to a steroid and the proteinuria almost normalized. We suggest that patients with primary SS accompanied by IgAN and MCD should receive corticosteroids as the first-line treatment.

The limitation in our case is that we could not completely exclude primary IgAN. Some reports suggest that **s**taining for galactose-deficient IgA1 is meaning tool for differential diagnosis between primary and secondary IgAN^[18,19]^ although there was recent report that the staining for galactose-deficient IgA1 could not differenciate secondary IgAN and from primary IgAN.^[20,21]^ This staining is not routinely performed in our hospital because of absence of antibody (KM 55 monoclonal antibody). However, given that her sicca symptoms occurred much earlier than renal abnormalities and there was no urinary abnormality in previous routine checks, it seems likely that IgAN in our case might be secondary to primary SS.

## 5. Conclusion

We additionally reported IgA with MCD associated with primary SS. Although tubulointerstitial lesions are common in primary SS as renal manifestations, we should suspect IgA with MCD when patient with SS showed hematuria, proteinuria, and clinically nephrotic syndrome, and perform renal biopsy for pathologically diagnosis and appropriate treatment. Our case suggests that IgAN with MCD may be characterized by a unique form of renal involvement in patients with primary SS; the condition may be viewed as a dual glomerulopathy and should respond to steroid treatment.

## Author contributions

**Conceptualization:** Jungyoon Choi, Dong Jun Park.

**Data curation:** Jungyoon Choi, Ha Nee Jang.

**Formal analysis:** Tae Won Lee, Eunjin Bae, Hyo Jung An.

**Investigation:** Ha Nee Jang.

**Methodology:** Jungyoon Choi, Hyo Jung An.

**Supervision:** Se-Ho Chang, Dong Jun Park.

**Validation:** Tae Won Lee, Eunjin Bae.

**Writing – original draft:** Jungyoon Choi.

**Writing – review & editing:** Se-Ho Chang, Dong Jun Park.
